# DEGRO consensus framework for undergraduate radiation therapy teaching in Germany: a white paper with integrated guidance on artificial intelligence in medical education

**DOI:** 10.1007/s00066-026-02575-4

**Published:** 2026-07-22

**Authors:** Philipp Linde, Biney Pal Singh, Maria Neu, Johannes Gollrad, Martin Bischoff, Gustavo R. Sarria, Annett Linge, Nanna E. Wielenberg, Anna-Lena Hillebrecht, Laura Höng, Martin Leu, Jörg Andreas Müller, Lukas Fabian Boehme, Anne Caroline Knöchelmann, Jan-Niklas Becker, Klaus Herfarth, Sonia Drozdz, Matthias Mäurer, Ina Patties, Daniel Fleischmann, Daniel Schmottermeyer, Alexander Fabian, Bernd Frerker, Sebastian Heß, Michael Oertel, Hendrik Dapper

**Affiliations:** 1 Department of Radiation Oncology, Cyberknife and Radiation Therapy, University Hospital of Cologne, Kerpener St 62, 50937 Cologne, Germany; 2 Department of Radiation Oncology, RWTH Aachen University Hospital, Pauwelsstraße 30, 52074 Aachen, Germany; 3 Department of Radiotherapy and Radiation Oncology, Klinik für Strahlentherapie und Radioonkologie, Faculty of Medicine, University of Augsburg, Stenglinstr. 2, 86156 Augsburg, Germany; 4 Department of Radiation Oncology, Charité—Universitätsmedizin Berlin, Berlin, Germany; 5 Department of Radiation Oncology, University Hospital of Ruhr-Universität Bochum, Marien Hospital Herne, Herne, Germany; 6 Department of Radiation Oncology, University Hospital Bonn, University of Bonn, Bonn, Germany; 7 Department of Radiotherapy and Radiation Oncology, Faculty of Medicine and University Hospital Carl Gustav Carus, TUD Dresden Technical University, Dresden, Germany; 8 Department of Radiation Oncology, Medical Center—University of Freiburg, Faculty of Medicine, University of Freiburg, Freiburg, Germany; 9 Department of Prosthetic Dentistry, Center for Dental Medicine, Medical Center—University of Freiburg, Faculty of Medicine, University of Freiburg, Hugstetterstr. 55, 79106 Freiburg, Germany; 10 Department of Radiation Oncology, Justus-Liebig-University Giessen, Giessen-Marburg University Hospital, Giessen, Germany; 11 Department of Radiotherapy and Radiation Oncology, University Medical Center, Göttingen, Germany; 12 Department of Radiation Oncology, University Hospital Halle, Halle (Saale), Germany; 13 Department of Radiotherapy and Radiation Oncology, University Medical Center Hamburg-Eppendorf, Hamburg, Germany; 14 Department of Radiotherapy, Hannover Medical School, Carl-Neuberg-Str. 1, 30625 Hannover, Germany; 15 Department of Radiation Oncology, University of Heidelberg, Heidelberg, Germany; 16 Department of Radiation Oncology, Jena University Hospital, Jena, Germany; 17 Department of Radiation Oncology, University Leipzig, Stephanstraße 9a, 04103 Leipzig, Germany; 18 Department of Radiation Oncology, LMU University Hospital, LMU Medizin, LMU Munich, Munich, Germany; 19 Department of Radiation Oncology, Klinikum rechts der Isar, Technische Universität München, Munich, Germany; 20 Department of Radiation Oncology, University Hospital Schleswig-Holstein, Kiel, Germany; 21 Department of Radiation Oncology, Rostock University Medical Center, 18059 Rostock, Germany; 22 Department of Radiation Oncology, University Hospital Würzburg, Julius-Maximilians-University, Würzburg, Germany; 23 Department of Radiation Oncology, University Hospital Münster, Münster, Germany

**Keywords:** Competency-based teaching, Digital learning, Curriculum, National competency-based learning objectives catalogue in medicine, Innovative teaching concepts

## Abstract

**Background and purpose:**

Undergraduate medical education is increasingly competency-based, digital, and interprofessional in accordance with the National Competence-Based Learning Objectives Catalogue for Medicine (Nationaler Kompetenzbasierter Lernzielkatalog Medizin, NKLM). Against this background, a nationally relevant, consensus-based framework for undergraduate radiation therapy education in Germany was developed by an expert panel of faculty representatives, aligned with the competencies expected at the end of the practical year (PJ) and in the final state examination context (M3), and complemented by guidance on the responsible educational use of artificial intelligence (AI).

**Methods:**

The framework was prepared by the German Society of Radiation Oncology (Deutsche Gesellschaft für Radioonkologie, DEGRO) working group “Medical Education” (AG Lehre), including a consolidated master document and 16 teaching portfolios that informed 15 candidate competencies. In a second step, 25 formally delegated representatives from 22 of the 36 university radiation oncology departments in Germany participated in a structured expert consensus process incorporating individual prioritization, small-group refinement, plenary consolidation, predefined voting thresholds, and postworkshop editorial integration.

**Results:**

The resulting framework comprised four components: (1) nine prioritized core competencies defining a national minimum standard at the PJ/M3 level; (2) implementation bandwidths (minimal, recommended, best practice) anchored in anonymized site-profile data; (3) a staged A/B/C assessment model aligned with the Miller pyramid; and (4) integrated AI guidance defining permitted use cases, nonnegotiable boundaries, AI literacy goals, and a practical educator-facing implementation logic. The tumor board was identified as a particularly suitable integrative teaching format.

**Conclusion:**

This white paper presents a nationally coordinated, competency-oriented framework for undergraduate radiation therapy education that combines a stable core with scalable local implementation options. By linking essential competencies, teaching bandwidths, staged assessment, and responsible AI guidance, greater consistency, coherence, and practical implementation of radiation therapy teaching across heterogeneous faculties is facilitated.

**Supplementary Information:**

The online version of this article (https://doi.org/10.1007/s00066-026-02575-4) contains supplementary material, which is available to authorized users.

## Introduction

Radiation therapy (RT) is a core component of modern medical care and is most prominently embedded in multimodal cancer treatment, where it contributes to curative, (neo-)adjuvant, and palliative treatment pathways across a wide range of oncological conditions [1, 2]. It is also relevant in selected benign indications and therefore represents an important and inherently interdisciplinary clinical field within undergraduate medical education, while its curricular representation often remains fragmented, with substantial variation in timing, depth, and teaching formats across institutions [3–5]. Establishing a shared educational core may therefore help reduce curricular fragmentation and strengthen the consistency of essential competencies in undergraduate medical training [6, 7].

Contemporary medical curricula increasingly emphasize competency-based, longitudinal, and clinically integrated learning [8–11]. In Germany, this development is reflected in the National Competence-Based Learning Objectives Catalogue for Medicine (Nationaler Kompetenzbasierter Lernzielkatalog Medizin, NKLM), which defines a national competency profile for medical graduates and serves as a reference framework for curriculum design, teaching, and assessment [12]. By shifting the emphasis from pure knowledge acquisition towards the development of clinical competencies, the NKLM aims to standardize core learning objectives across faculties while allowing local curricular flexibility and longitudinal integration. Within undergraduate medical education, this creates an opportunity to define a principled and teachable core in radiation therapy education (RTE) that is nationally applicable, aligned with competency-based education, and adaptable to heterogeneous local curricula [13–15].

Furthermore, over the past few years, a growing body of evidence has emerged on what constitutes meaningful undergraduate RTE, particularly in its oncological context, reflecting both expert perspectives and student expectations regarding scope, content, and integration within the medical curriculum [1, 4, 5, 13]. At the same time, generative artificial intelligence (AI) has rapidly become relevant in higher and medical education, including its use for learning support, content structuring, and assessment-related tasks [16, 17]. This development creates not only new educational opportunities, but also a need for explicit guidance on responsible use, transparency, validation, and AI literacy in undergraduate training.

Against this background, this white paper outlines the core competencies in RT that should be expected of all graduating medical students, irrespective of their later specialty choice. It proposes a nationally relevant, consensus-based framework for undergraduate RTE in Germany and integrates concise guidance on the responsible educational use of AI, including AI literacy goals and implementation-oriented recommendations for educators.

### Aim and scope of the white paper

This white paper aims to define a nationally relevant and locally adaptable minimum standard for undergraduate RTE in Germany, with oncology as the dominant clinical context. Its primary target group is medical students at the transition to the final undergraduate training phase, operationalized here as the level expected by the end of the practical year (PJ) and in the context of the final state examination (M3). The framework is therefore intentionally oriented toward clinical actionability rather than specialist depth.

The proposed framework does not seek to describe a specialist curriculum, define mandatory teaching hours, or prescribe a uniform national implementation model. Instead, it focuses on a manageable core of clinically relevant competencies related to treatment principles, referral and indication awareness, patient safety, recognition and initial management of radiotherapy-related side effects and oncologic emergencies, patient-centered communication, and interdisciplinary decision-making.

To ensure applicability across heterogeneous faculties, the scope is deliberately limited. The framework excludes detailed dosimetry, in-depth treatment planning, advanced equipment-specific expertise, and organ-specific comprehensiveness at specialist level. At the same time, it allows local curricula to extend beyond the core through additional modules, format-specific enrichment, and institution-specific implementation pathways.

## Methods

### Participants and composition of the expert panel

The structured expert consensus meeting was held in Berlin on 13–14 March 2026 under the auspices of the German Society of Radiation Oncology (Deutsche Gesellschaft für Radioonkologie e. V., DEGRO) working group “Medical Education” (DEGRO AG Lehre). All university radiation oncology departments in Germany were informed in advance and invited through a save-the-date notice, including announcement in the official DEGRO newsletter.

The expert panel comprised 25 formally delegated representatives from 22 of the 36 invited departments, including the chairs of the DEGRO working group “Medical Education”. Participants represented a broad range of clinical and academic career stages and held formal responsibility for undergraduate medical teaching at their institutions. Participating institutions can be identified from the author affiliations.

### Preparatory work

Preparatory work preceded the workshop. A consolidated master document was developed as the editorial working basis, integrating the draft white paper, workshop and moderation materials, an AI consensus component, and appendices.

This master version was explicitly described as a working and editorial version rather than a final manuscript. It combined a principle- and competency-oriented narrative core with operational workshop materials and was designed to keep the main text stable while assigning mapping and template-based elements to appendices and a versioned living appendix [18–20].

In advance, structured departmental submissions of teaching portfolios summarizing the local teaching formats, curricular integration, assessment approaches, and educational priorities in undergraduate RTE were collected. Sixteen portfolios submitted before the meeting were synthesized into an initial set of 15 candidate competencies. The preliminary candidate competencies were extracted on the basis of recurring topics, their relevance to the PJ/M3 level, their clinical and curricular importance, and their anticipated feasibility across heterogeneous faculties. The submitted portfolios did not correspond one-to-one to all represented departments, but served as structured feasibility and content anchors for the preparatory phase.

Additional preparatory materials included a site heatmap, a draft tumor board kit, a template for later NKLM mapping, a tool-disclosure form for the AI component, and standardized print materials for prioritization, group work, and plenary consolidation. These materials were intended to ensure that the workshop would start from a structured synthesis rather than from open-ended brainstorming [16]. All participants, including those from departments without a submitted portfolio, received the same preparatory materials in advance. Portfolio submission constituted the preparatory phase, but final prioritization and consensus decisions were determined through the structured workshop process.

**Fig. 1:**
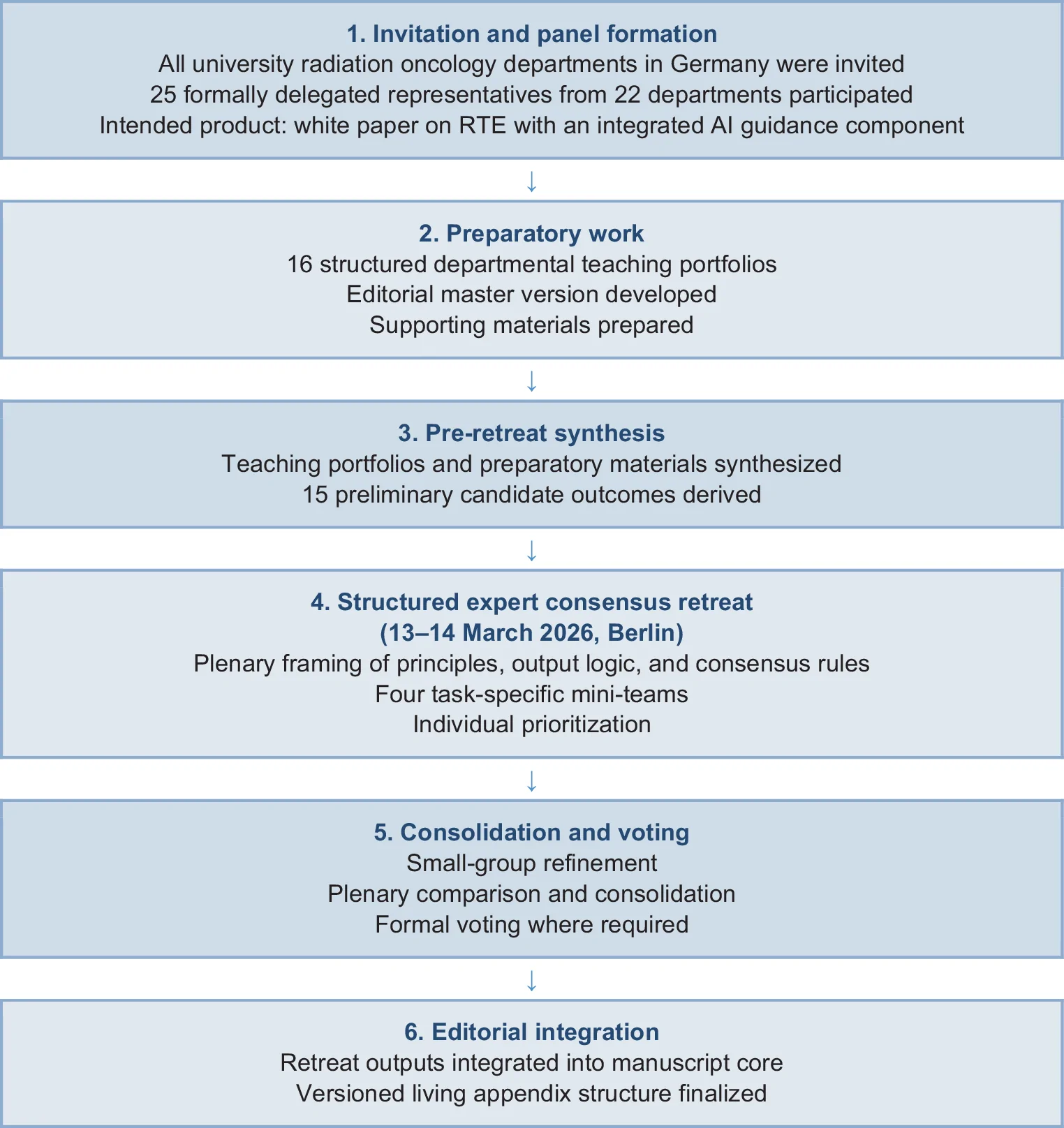
Flowchart of the development and consensus process of the white paper framework. *RTE* radiation therapy education

The development process is additionally illustrated in Fig. 1 and was documented with reference to recent reporting recommendations for consensus-based framework development [21].

### Workshop structure and consensus procedure

The structured expert consensus meeting followed a predefined structure of introductory framing, prioritization, small-group work, and plenary consolidation, guided by established principles of structured consensus development [16, 21–24]. Two main working phases (3 h each) were defined: the first focused on structuring and prioritization, whereas the second focused on harmonization and final consolidation. The overarching aim was to establish a shared working basis for subsequent manuscript development.

At the outset, participants were introduced to the white paper framework, output logic, and consensus rules, with a clear emphasis on producing editorial-ready outputs rather than open-ended ideas. Each phase was expected to yield visible structured outputs, such as summaries, templates, rubrics, checklists, or consensus pages. A stable narrative main text with a separately versioned living appendix was introduced as a central design principle.

The first working phase was conducted in four small groups: the first developed guiding statements, scope boundaries, and key messages; the second refined and prioritized candidate competencies and added NKLM anchors where feasible; the third developed a practice-oriented tumor board teaching package, including roles, workflow, and implementation profiles; and the fourth designed an assessment framework aligned with the Miller pyramid (A: written minimum assessments, ‘knows how’; B: Objective Structured Clinical Examination (OSCE) type formats, ‘shows how’; C: workplace-based assessments such as mini-clinical evaluation exercise (Mini-CEX), ‘does’) [25].

Predefined roles within each group included moderation, timekeeping, reporting, and evidence stewardship. The evidence steward ensured traceability of outputs to source materials such as mapping tools, site matrices, tumor board literature, or national reference documents [16].

Open questions arising from the first phase were converted into formulation-ready statements and addressed during the second working phase in plenary consolidation, followed by assignment of responsibilities and handover to the editorial group.

### Prioritization and consolidation [17]

The competence framework initially drew on 15 candidate competencies synthesized from the submitted teaching portfolios and preparatory drafting work. During the workshop, these competencies were reviewed using an explicit distinction between must-have and nice-to-have elements. Must-have items were defined as mandatory, nationally relevant minimum standards at the PJ/M3 level that were clinically important, educationally meaningful, and in principle teachable and assessable across heterogeneous faculties. Nice-to-have items were considered valuable but more local, resource-dependent, or format-dependent and could therefore remain facultative but visible without becoming part of the core standard.

Prioritization combined individual voting, structured small-group refinement, and plenary consolidation. In an initial step, participants completed an individual dot-voting exercise using ten points per person to indicate priorities among the 15 candidate competencies. On the following day, these prioritization signals were compared with the competency set developed by the small group and jointly considered in plenary.

The dot-voting exercise served to structure and weight the initial prioritization, but did not by itself determine the final framework. Final inclusion in the manuscript core was established through plenary consolidation and, where required, formal voting. In this process, the 15 preliminary candidate competencies were reduced to nine prioritized core competencies, while additional items were reassigned to extension material, optional modules, or the appendix. Where formal voting was required to confirm plenary decisions, all participating delegated representatives were eligible to vote, and agreement consistently reached the predefined consensus threshold of ≥ 75%.

The same prioritization logic was also applied to broader framework elements such as the tumor board package, the implementation bandwidth model, the staged assessment blueprint, and the integrated AI guidance. Across all work phases, outputs were expected to be formulated as observable, teachable, and assessable behaviors rather than as broad thematic headings [26].

Accordingly, plenary consolidation was intended not to complete the final manuscript on site, but to produce a consolidated set of decisions, artefacts, and handover notes for subsequent editorial integration.

### Postworkshop editorial process

The workshop was explicitly framed as the development of a consensus-based national statement and an editorial foundation for subsequent manuscript preparation. Postworkshop drafting was expected to build on workshop-approved artefacts and handover notes rather than to reopen foundational consensus questions. Editorial coordination and review were used to consolidate the retreat outputs into a stable manuscript core and an updateable appendix structure. In methodological terms, the process should be understood as a structured expert consensus development approach rather than as a formal Delphi study. The reporting of the framework development process was guided by an explicit commitment to transparent documentation of preparatory synthesis, consensus procedures, and postworkshop editorial integration, in line with recent recommendations for reporting competency framework development in the health professions [27].

### Ethical standards

Ethics approval was not required for this consensus-based educational development process.

## Results

The present work describes a consensus-based framework for undergraduate RTE, with a specific focus on the transition to the PJ/M3 level. The framework comprises four complementary components: a prioritized core competency set, implementation-oriented teaching formats with defined bandwidths, a staged assessment model, and integrated AI guidance. This structure reflects the central design principle of distinguishing clearly between a nationally defined core and locally adaptable extensions, while ensuring feasibility across heterogeneous institutional settings.

### A consensus-based core framework for undergraduate radiation therapy education

#### Guiding statement and key messages

Undergraduate medical students should understand radiation therapy as the patient-centered, precise use of ionizing radiation for cure or symptom relief and as a central pillar of interdisciplinary cancer care, sustained by teamwork and communication.

Within this framework, RT is presented as a clinical discipline with broad availability, an essential pillar of cancer care for more than half of all patients with cancer, interdisciplinary, interprofessional, and innovative, noninvasive, and longitudinally accompanying patients across the treatment trajectory.

#### Nine prioritized core competencies

The framework comprises nine prioritized core competencies at the PJ/M3 level. These competencies define the proposed national minimum standard, whereas additional content was deliberately assigned to extension material, optional modules, or appendices. Taken together, the nine core competencies span conceptual understanding, clinical actionability, patient-centered communication, and interdisciplinary collaboration. Core competencies are summarized in Table 1.

**Table 1:** Nine prioritized core competencies for undergraduate radiation therapy education at the PJ/M3 level

No.	Prioritized core competency	Expected observable performance at the PJ/M3 level
1	Explain radiation therapy as a core treatment pillar	Explains what radiation therapy is and describes its indications in curative and symptom-oriented (palliative) multimodal cancer care, as well as in benign conditions.
2	Identify patients who require radiation therapy within multimodal care pathways	Recognizes typical clinical situations in which radiation therapy input is indicated and identifies and explains the relevant professional interfaces within multimodal care pathways.
3	Provide patient-centered benefit–risk communication and basic informed counselling	Elicits patient preferences and provides a structured, patient-centered explanation of expected benefits, risks, and next steps.
4	Describe the radiation therapy workflow and basic AI-related integration	Describes the basic workflow of radiotherapy planning and delivery and relates potential AI applications to this workflow in a clinically meaningful way.
5	Recognize and initially manage common acute radiotherapy toxicities	Identifies red flags and proposes initial management steps for common acute treatment-related toxicities.
6	Outline late effects, prevention, and follow-up logic	Names typical possible late effects and relates them to the expected benefits of radiotherapy.
7	Recognize and prioritize oncologic emergencies with relevance to radiotherapy	Demonstrates awareness of oncologic emergencies with potential radiotherapy relevance and prioritizes appropriate next steps.
8	Explain palliative treatment options including radiotherapy	Explains the role of radiotherapy across palliative indications, from prophylactic to symptom-oriented settings.
9	Formulate interdisciplinary oncological treatment recommendations involving radiotherapy	Formulates a concise interdisciplinary treatment recommendation involving radiotherapy and justifies it with reference to the clinical context and underlying evidence.

Detailed NKLM mapping was deliberately assigned to the versioned living appendix in order to preserve the stability and readability of the manuscript core. Additional content beyond the core framework was deliberately retained as extension material, optional modules, or appendix content.

### Teaching formats and implementation bandwidths

The framework does not prescribe fixed teaching hours or a single mandatory delivery format. Instead, it defines implementation bandwidths that allow local adaptation while preserving a shared national core. Across sites, lectures and small group teaching were broadly represented, patient contact was common, whereas digital formats, tumor board components, and OSCE/Objective Structured Practical Examination (OSPE)- or workplace-based assessment formats were more heterogeneously implemented. Accordingly, the framework adopts an competency- and logic-based model with defined implementation bandwidths rather than a fixed-hour approach. Table 2 summarizes the cross-site teaching profiles underlying the implementation bandwidth model.

**Table 2:** Anonymized site-profile matrix underlying the implementation bandwidth model.

Site	Lecture	Seminar/small-group teaching	Patient contact/bedside teaching	Digital formats	Rotation/practical attachment	Tumor board elements	OSCE/OSPE	Examination	PJ integration
A	▪	▪	▪	▪	▪	▪	□	▪	▪
B	▪	▪	▪	□	▪	□	□	□	▪
C	▪	▪	▪	□	□	□	□	□	▪
D	▪	▪	▪	□	▪	□	▪	▪	▪
E	▪	▪	▪	□	□	□	□	□	▪
F	▪	▪	□	□	□	◐	□	▪	◐
G	▪	▪	▪	▪	▪	□	□	▪	▪
H	▪	▪	▪	◐	▪	▪	□	▪	▪
I	▪	▪	◐	▪	▪	◐	□	□	◐
J	▪	▪	▪	▪	□	□	□	▪	◐
K	▪	▪	▪	□	□	□	▪	▪	▪
L	▪	▪	▪	□	▪	▪	▪	▪	▪
M	◐	▪	▪	□	▪	□	□	▪	▪
N	▪	▪	◐	□	□	□	□	□	◐
O	▪	▪	◐	□	□	▪	□	▪	▪
P	▪	▪	▪	□	▪	□	□	□	▪

▪ = clearly represented, ◐ = partly represented/optional, □ = not reported in the submitted portfolio. Coding was conservative and based on explicit mentions in the submitted portfolios.

#### Implementation bandwidths: minimal, recommended, best practice

Accordingly, the framework distinguishes three implementation bandwidths—minimal, recommended, and best practice (Table 3). The minimal level represents a nationally feasible baseline with low-threshold, widely transferable formats. The recommended level adds structured clinical and interactive elements, including patient contact and tumor board-related teaching where feasible. The best-practice level comprises more resource-intensive formats, such as simulation-based tumor board approaches, workplace-based assessments, and broader curricular integration.

**Table 3:** Implementation bandwidths for undergraduate radiation therapy education

Bandwidth	Core components	Assessment mix	Portfolio-based feasibility examples	Consensus rationale
Minimal	Case-based core seminars, one introductory lecture, and radiation protection microlearning	Level A	Lecture-based and seminar formats were represented across all anonymized site profiles	Low-threshold and feasible as a starting point across institutions
Recommended	Minimal profile *plus* one structured patient contact and one tumor board simulation embedded in a seminar format	Level A plus optional B	Patient contact was common across portfolios; tumor board elements were present at selected sites and could serve as transferable model components	Maximizes educational gain with moderate additional effort
Best practice	Rotation or practical attachment, hybrid tumor board format, OSCE/OSPE, and Mini-CEX during the practical year (PJ)	Levels A + B + C	OSCE/OSPE and workplace-based assessments were minority formats in the site profiles and therefore represent realistic aspirational targets rather than baseline expectations	Suitable as an aspirational curriculum model without redefining the national minimum standard

The implementation bandwidth model was anchored in anonymized site profiles and was intended to support feasibility-oriented local adaptation rather than institutional ranking or fixed national teaching-hour requirements.

Each bandwidth is linked to a corresponding assessment mix, with Level A representing the minimum national expectation and Levels B and C indicating optional developmental extensions. Overall, the model provides a feasible implementation scaffold across heterogeneous institutional settings.

#### The tumor board as an integrative teaching format

Within the framework, the tumor board is positioned as a particularly suitable integrative teaching format. It is centered on the learning objective that students should be able to explain and critically appraise interdisciplinary decision-making processes and understand the function and workflow of a tumor board. Its didactic strength lies in integrating clinical reasoning, evidence-based decision-making, communication, teamwork, and patient-related trade-offs within a single setting. The teaching package is structured around defined participant roles and corresponding quality criteria, including moderated consensus-building, structured case presentation, consequence-oriented interdisciplinary input, and explicit attention to therapeutic goals, supportive care, and treatment limitations. Across implementation bandwidths, the tumor board is operationalized in a scalable manner, ranging from introductory simulation-based sessions to combined observation-and-simulation formats and advanced formats including real-board participation, student-led discussions, and structured reflection.

At the recommended implementation level, for example, a tumor board session may be structured around a simulated case. In this case, students would receive a brief summary of the case, assume different professional perspectives, and discuss multimodal treatment options under guided moderation. This format can operationalize core competencies such as referral awareness, interdisciplinary decision-making, and patient-centered benefit–risk communication. An illustrative example is provided in Supplementary Material 2.

### Assessment framework

The framework includes a staged assessment model that aligns the national minimum standard with heterogeneous local resources and curricular maturity. Rather than defining a single mandatory assessment format, the framework distinguishes between a universally feasible minimum level and optional developmental extensions. This logic prevents assessment formats from becoming a precondition for the curricular core.

#### The A/B/C model

The assessment model is structured as a three-level A/B/C framework aligned with the Miller pyramid [22]. Level A targets applied knowledge (“knows how”) and comprises written formats such as short-answer or case-based questions. Level B targets observable performance (“shows how”) and includes OSCE-type formats (e.g., counselling or structured case presentation). Level C targets performance under supervision (“does”) and comprises workplace-based assessment formats such as Mini-CEX or related workplace-based assessment (WBA) approaches (Table 4).

**Table 4:** Staged assessment framework for undergraduate radiation therapy education

Level	Target (Miller)	Typical formats	Resource profile	Minimum quality requirement
A	Knows how	Short answer questions, key-feature questions, brief case-based multiple-choice items	Low	Competency-specific blueprint and model answer
B	Shows how	OSCE-type formats, e.g., counselling, toxicity management, structured case presentation	Moderate	Scoring rubric and examiner briefing
C	Does (under supervision)	Mini-CEX/WBA in simulation, observation, or clinical placement	Variable	Mandatory feedback and entrustment-oriented rating

Table 4 summarizes the staged assessment model adopted in the framework.

#### Minimum assessment expectations and developmental pathways

Level A defines the minimum national assessment standard, with each core competency linked to at least one feasible A‑level format. Levels B and C are not part of the national minimum standard, but represent optional developmental extensions depending on local curricular maturity, patient access, and available resources. This approach ensures broad feasibility while allowing more advanced, practice-oriented assessment formats where appropriate.

The framework also includes exemplar formats (e.g., structured short-answer questions, OSCE stations, and Mini-CEX/WBA) to illustrate the intended depth of assessment and to support local implementation rather than mandatory templates.

#### Alignment of competencies, teaching formats, and assessment

Assessment is embedded within a constructive alignment framework, in which learning competencies define expected competencies, teaching formats support their development, and assessment renders achievement observable. Accordingly, the framework is competency-driven rather than format-driven. Teaching and assessment are coherently linked through the same implementation bandwidth logic: low-threshold written formats ensure a feasible minimum standard, while OSCE-type and workplace-based assessments provide increased observational depth where local conditions permit.

### Integrated guidance on AI in radiation therapy education

The framework includes integrated guidance on the responsible educational use of generative artificial intelligence (AI) in RTE. This component is not framed as a stand-alone technology paper, but as a compact governance and implementation element within the white paper itself. Its purpose is to define appropriate use under conditions, identify clear nonnegotiable boundaries, specify core AI literacy goals, and provide a practical orientation for educators.

#### Core principle, use cases, and nonnegotiable boundaries

Within this framework, generative AI is understood as both a teaching and learning aid and an explicit learning object. At the same time, it is not regarded as a substitute for human responsibility, disciplinary expert review, or assessment governance. Permitted use cases under explicit conditions include formative case-vignette drafting, learning support for structuring and summarizing content, AI-assisted drafting of assessment items or rubrics subject to human blueprinting and quality control, and AI literacy itself as an explicit teaching goal. Across all permitted uses, human review, transparency, and source- and plausibility-checking remain nonnegotiable.

Nonnegotiable boundaries were defined with equal clarity. These include the prohibition of entering personal patient data into generative AI tools, the use of unvalidated medical content without source-checking and contextual review, the absence of transparent disclosure where relevant, and any weakening of format-specific assessment integrity or human final responsibility in teaching and assessment decisions.

#### AI literacy and practical implementation

AI literacy was explicitly integrated as a small but distinct competency block within the framework. Students are expected to understand opportunities and limitations of generative AI in clinical contexts, identify hallucinations and bias and apply validation strategies, implement data protection and transparency in practice, and reflect on AI use in ways that consciously address risks such as deskilling. For educators, the guidance is operationalized through a deliberately simple implementation rule: before using AI in teaching, it should be asked whether the data are nonsensitive, whether the use is transparent, and whether disciplinary validation is secured. In this way, the AI component extends beyond permission logic and functions as both a governance statement and an educator-facing implementation aid. The core elements of the integrated AI guidance are summarized in Table 5.

**Table 5:** Integrated AI guidance in radiation therapy education

*Core principle:* AI is a teaching/learning aid and learning object, not a substitute for human responsibility.
*Permitted under conditions:* formative case-vignette drafts, learning support, AI-assisted assessment drafting, explicit AI-literacy teaching.
*Nonnegotiable boundaries:* no patient data, no unvalidated medical content, clear transparency rules, format-specific assessment integrity, human final responsibility.
*Operational rule:* check data sensitivity, transparency, and disciplinary validation before any educational use.

## Discussion

This white paper establishes, for the first time, a consensus-based, competency-oriented framework for undergraduate RTE in Germany with a specific focus on the transition to the PJ/M3 level. Rather than prescribing a specialist curriculum or fixed teaching hours, it operationalizes competency-based medical education in a discipline-specific context and provides a feasible model for implementation across diverse institutional settings. This is particularly relevant in Germany, where teaching structures and curricular formats vary across faculties and are developed locally, while the NKLM increasingly functions as a central national competency-based reference framework.

The framework is educationally relevant because it aligns with the broader shift toward competency-based, competency-oriented medical education and addresses a discipline-specific challenge: despite its central role in multimodal cancer care, radiation therapy remains unevenly represented in undergraduate curricula and is not prominently consolidated within national competency frameworks [4, 11, 12, 28, 29]. It therefore seeks to strengthen the consistency and curricular visibility of core competencies while preserving local flexibility rather than enforcing a uniform national delivery model.

### Educational rationale and implementation value

A strength is the deliberate use of implementation bandwidths instead of a uniform national delivery model. This aligns with international standards such as the World Federation for Medical Education, which emphasize adaptability to local context over rigid curricular prescriptions [26]. The framework derives implementation value from its constructive-alignment logic [30]. In competencies-based curriculum design, intended learning competencies should guide both teaching activities and assessment rather than the reverse [31]. By linking core competencies to teaching bandwidths, to a staged A/B/C assessment model, and best-practice profiles, the framework maintains this alignment while remaining feasible across heterogeneous institutional settings [32].

Finally, the framework has practical implementation value because it combines a stable narrative core with selectively updateable operational elements. This reduces the risk that local implementation debates become blocked by catalogue detail, evolving mapping requirements, or format-specific ideal models. Instead, the paper offers a core that is stable enough for national orientation and flexible enough for local curricular translation. That combination is particularly important in a field such as radiation therapy, whose educational relevance is high, while its curricular representation and implementation opportunities continue to vary across institutions [5, 13, 33].

### Tumor board and AI as integrative components

Two integrative elements are particularly characteristic of the framework: the tumor board and the AI guidance component. The tumor board is educationally valuable because it integrates clinical reasoning, interprofessional communication, evidence-informed decision-making, and patient-related trade-offs within a single learning setting [34, 35]. This aligns with literature on interprofessional education and with emerging evidence that simulated, shadowing-based, or student-oriented tumor board formats enhance understanding of interdisciplinary workflows and treatment recommendation processes [36–38]. From a learner perspective, the format addresses a common gap in fragmented oncology teaching by making expert reasoning and multimodal decision-making visible in a scalable way across different curricular contexts [39, 40]. The framework’s emphasis on scalable formats—ranging from simulation to observation—supports access across different curricular contexts while preserving educational value [41, 42]. Reports suggest that this kind of exposure can change students’ perceptions of collaboration and strengthen their understanding of how treatment recommendations are actually generated [43, 44].

The AI component plays a complementary integrative role. Rather than treating AI as a stand-alone topic, the framework embeds it as both a practical educational issue and a defined literacy domain [45]. This is educationally defensible and policy-aligned [46, 47]. European guidance on trustworthy AI and on AI and data use in education places strong emphasis on transparency, human oversight, privacy, accountability, and context-sensitive use by educators [48]. In a German academic context, closely related principles are reflected in guidance from the German Research Foundation, which stresses that generative models do not relieve users of content-related or formal responsibility and that appropriate disclosure remains essential [49, 50]. These principles map closely onto the framework’s own nonnegotiable boundaries and educator-facing implementation logic [51].

By defining explicit AI literacy goals, the framework avoids treating AI primarily as an efficiency tool and acknowledges that students need more than operational permission to use generative tools: they need to understand limitations, identify hallucinations and bias, apply validation strategies, and reflect on risks such as deskilling [52–55]. This is particularly relevant in RTE, where digital technologies are highly visible and where students may otherwise overestimate the authority or reliability of AI-generated outputs. This combined emphasis links innovation to governance, and practical use to critical reflection [56].

### Strengths and limitations

A major strength of the framework lies in its combination of national coordination with practical curricular feasibility. The consensus process was based on formally delegated representatives, structured preparatory work, and defined consensus procedures, thereby providing a robust foundation for a nationally coordinated position statement while maintaining a clear focus on implementable competencies. The process was further informed by established principles of structured consensus development [19–21].

A further strength lies in the framework’s resilience to changes in national competency catalogues. Although aligned with the NKLM, it was formulated at the level of outcome logic rather than specific catalogue identifiers. This allows the core manuscript to remain stable, with updates confined to a versioned appendix, thereby maintaining alignment without overdependence on evolving catalogue detail.

Several limitations should be acknowledged. First, the process does not constitute a formal Delphi study, but rather a structured consensus approach combining workshop-based prioritization with editorial consolidation. Second, although 22 departments were represented, this should not be interpreted as full national coverage. Third, the site-profile matrix and teaching portfolios served as feasibility anchors rather than as a comprehensive national curriculum survey.

In addition, the framework was developed primarily by experts in the discipline and did not include formal student co-design. Future iterations should ideally incorporate structured student involvement to complement the expert-based consensus process [57].

The framework has not yet undergone prospective validation across institutions, and data on implementation, acceptability, and educational effectiveness are currently lacking. Future refinement may explore closer alignment with entrustment-oriented approaches, while the AI guidance component will likely require iterative refinement as evidence and regulatory frameworks continue to evolve [58].

### Implications for national and local curriculum development

At the national level, the framework may serve as a reference for harmonization without imposing curricular uniformity. In the German context, where the NKLM provides a central competency-based framework but institutional structures and teaching formats vary, it helps define the role of RT in undergraduate medical education while allowing local adaptation within predominantly oncological teaching contexts.

At the local level, the framework is best understood as a scaffold for curriculum development rather than as a fixed curriculum. Faculties can use the core competencies as a starting point, apply the bandwidth model to define feasible entry levels, and align teaching and assessment stepwise. In this way, the framework provides not only competencies, but also a practical structure for prioritization, phased implementation, and curricular translation.

A further implication concerns faculty development. Competency-based frameworks require educators who can translate competencies into teaching, feedback, and assessment. This is particularly relevant for formats such as tumor boards, workplace-based assessments, and AI-related teaching. Accordingly, successful implementation will depend on targeted support for educators, including assessment literacy, practical guidance, and structured feedback loops.

Finally, implementation of the framework may strengthen the quality and coherence of undergraduate RTE, enhance student engagement with the field, and support longer-term workforce development as a secondary effect.

## Conclusion

This white paper presents a consensus-based, competency-oriented framework for undergraduate radiation therapy (RT) education in Germany that is nationally relevant and locally adaptable. Focused on the practical year/final state examination (PJ/M3) level, it defines a proposed national minimum standard through nine prioritized core competencies, complemented by implementation bandwidths, a staged assessment model, and integrated guidance on the responsible use of artificial intelligence (AI) in education.

By combining a stable core with scalable implementation options, the framework aims to enhance the consistency and feasibility of RT teaching across heterogeneous faculties while preserving curricular flexibility. It may also strengthen student awareness of and engagement with the field, thereby supporting longer-term interest in RT and related clinical careers.

## Supplementary Information

ESM1: Supplementary material 1

## Data Availability

The datasets generated and analyzed during the current study are available from the corresponding author on reasonable request.

## Abbreviations

AI: Artificial intelligence
DEGRO: German Society for Radiation Oncology; Deutsche Gesellschaft für Radioonkologie
M3: Final state examination
Mini-CEX: Mini-clinical evaluation exercise
NKLM: Nationaler Kompetenzbasierter Lernzielkatalog Medizin
OSCE: Objective Structured Clinical Examination
OSPE: Objective Structured Practical Examination
PJ: Practical year; Praktisches Jahr
RT: Radiation therapy
RTE: Radiation therapy education
WBA: Workplace-based assessment
