# Correction to: DEGRO consensus framework for undergraduate radiation therapy teaching in Germany: a white paper with integrated guidance on artificial intelligence in medical education

**DOI:** 10.1007/s00066-026-02603-3

**Published:** 2026-09-04

**Authors:** Philipp Linde, Biney Pal Singh, Maria Neu, Johannes Gollrad, Martin Bischoff, Gustavo R. Sarria, Annett Linge, Nanna E. Wielenberg, Anna-Lena Hillebrecht, Laura Höng, Martin Leu, Jörg Andreas Müller, Lukas Fabian Boehme, Anne Caroline Knöchelmann, Jan-Niklas Becker, Klaus Herfarth, Sonia Drozdz, Matthias Mäurer, Ina Patties, Daniel Fleischmann, Daniel Schmottermeyer, Alexander Fabian, Bernd Frerker, Sebastian Heß, Michael Oertel, Hendrik Dapper

**Affiliations:** 1 Department of Radiation Oncology, Cyberknife and Radiation Therapy, University Hospital of Cologne, Kerpener St 62, 50937 Cologne, Germany; 2 Department of Radiation Oncology, RWTH Aachen University Hospital, Pauwelsstraße 30, 52074 Aachen, Germany; 3 Department of Radiotherapy and Radiation Oncology, Klinik für Strahlentherapie und Radioonkologie, Faculty of Medicine, University of Augsburg, Stenglinstr. 2, 86156 Augsburg, Germany; 4 Department of Radiation Oncology, Charité—Universitätsmedizin Berlin, Berlin, Germany; 5 Department of Radiation Oncology, University Hospital of Ruhr-Universität Bochum, Marien Hospital Herne, Herne, Germany; 6 Department of Radiation Oncology, University Hospital Bonn, University of Bonn, Bonn, Germany; 7 Department of Radiotherapy and Radiation Oncology, Faculty of Medicine and University Hospital Carl Gustav Carus, TUD Dresden Technical University, Dresden, Germany; 8 Department of Radiation Oncology, Medical Center—University of Freiburg, Faculty of Medicine, University of Freiburg, Freiburg, Germany; 9 Department of Prosthetic Dentistry, Center for Dental Medicine, Medical Center—University of Freiburg, Faculty of Medicine, University of Freiburg, Hugstetterstr. 55, 79106 Freiburg, Germany; 10 Department of Radiation Oncology, Justus-Liebig-University Giessen, Giessen-Marburg University Hospital, Giessen, Germany; 11 Department of Radiotherapy and Radiation Oncology, University Medical Center, Göttingen, Germany; 12 Department of Radiation Oncology, University Hospital Halle, Halle (Saale), Germany; 13 Department of Radiotherapy and Radiation Oncology, University Medical Center Hamburg-Eppendorf, Hamburg, Germany; 14 Department of Radiotherapy, Hannover Medical School, Carl-Neuberg-Str. 1, 30625 Hannover, Germany; 15 Department of Radiation Oncology, University of Heidelberg, Heidelberg, Germany; 16 Department of Radiation Oncology, Jena University Hospital, Jena, Germany; 17 Department of Radiation Oncology, University Leipzig, Stephanstraße 9a, 04103 Leipzig, Germany; 18 Department of Radiation Oncology, LMU University Hospital, LMU Medizin, LMU Munich, Munich, Germany; 19 Department of Radiation Oncology, Klinikum rechts der Isar, Technische Universität München, Munich, Germany; 20 Department of Radiation Oncology, University Hospital Schleswig-Holstein, Kiel, Germany; 21 Department of Radiation Oncology, Rostock University Medical Center, 18059 Rostock, Germany; 22 Department of Radiation Oncology, University Hospital Würzburg, Julius-Maximilians-University, Würzburg, Germany; 23 Department of Radiation Oncology, University Hospital Münster, Münster, Germany

**Correction to:**

**Strahlenther Onkol 2026**

https://doi.org/10.1007/s00066-026-02575-4

In this article the “Conflict of interest” statement was incorrectly given as “A. Fabian received honoraria from Merck Sharp and Dohme (MSD) as well as a research award from Lilly Germany Foundation. P. Linde, B. Pal Singh, M. Neu, J. Gollrad, M. Bischoff, G.R. Sarria, A. Linge, N.E. Wielenberg, A.-L. Hillebrecht, L. Höng, M. Leu, J.A. Müller, L.F. Boehme, A.C. Knöchelmann, J.-N. Becker, K. Herfarth, S. Drozdz, M. Mäurer, I. Patties, D. Fleischmann, D. Schmottermeyer, B. Frerker, S. Heß, M. Oertel and H. Dapper declare that they have no competing interests.” but should have been “A. Fabian received honoraria from Merck Sharp and Dohme (MSD) as well as a research award from Lilly Germany Foundation. P. Linde received honoraria for invited lectures from MSD as well as from Merck KGaA, Darmstadt, Germany. The authors declare that they have no competing interests relevant to the content of this article. B. Pal Singh, M. Neu, J. Gollrad, M. Bischoff, G.R. Sarria, A. Linge, N.E. Wielenberg, A.-L. Hillebrecht, L. Höng, M. Leu, J.A. Müller, L.F. Boehme, A.C. Knöchelmann, J.-N. Becker, K. Herfarth, S. Drozdz, M. Mäurer, I. Patties, D. Fleischmann, D. Schmottermeyer, B. Frerker, S. Heß, M. Oertel and H. Dapper declare that they have no competing interests.”.

The original article has been corrected.

