# Supplementary material for: DEGRO consensus framework for undergraduate radiation therapy teaching in Germany: a white paper with integrated guidance on artificial intelligence in medical education
Source: Strahlenther Onkol. 2026 Jul 22;202(10):1016–28. doi: 10.1007/s00066-026-02575-4 (PMC13597594; doi:10.1007/s00066-026-02575-4)
Supplement: Supplementary file 1 [file 66_2026_2575_MOESM1_ESM.docx]

**Supplementary Material 1-5**

**Supplementary Material 1. Implementation bandwidths for undergraduate radiation oncology education**

**Purpose**
This implementation aid summarizes the three implementation bandwidths proposed in the white paper for undergraduate radiation oncology education: **minimal**, **recommended**, and **best practice**. It is intended to support local curriculum planning by linking the national core framework to scalable, context-sensitive implementation options.

**Core principle**
The framework does not prescribe fixed national teaching hours or a single mandatory delivery format. Instead, it offers a bandwidth model that preserves a shared national core while allowing faculties to adapt implementation to local curricular structures, staffing, and resources.

**Minimal implementation profile**
The minimal profile represents a nationally feasible baseline. It includes:

- **case-based core seminars**
- **one introductory lecture**
- **radiation protection microlearning**

Assessment remains at **Level A**, using low-threshold written formats aligned with the core outcomes. This profile is intended as a realistic entry level for institutions seeking broad feasibility and low implementation barriers.

**Recommended implementation profile**
The recommended profile builds on the minimal level by adding:

- **one structured patient contact**
- **one tumor board simulation embedded in a seminar format**

Assessment includes **Level A plus selected optional B-level formats** where feasible. This profile is intended to maximize educational gain with moderate additional effort and stronger clinical integration.

**Best-practice implementation profile**
The best-practice profile extends the framework through:

- **a rotation or practical attachment**
- **a hybrid tumor board format**
- **OSCE/OSPE elements**
- **Mini-CEX during the practical year (PJ)**

Assessment integrates the full **A/B/C model**. This profile is not defined as a national minimum requirement, but as a realistic aspirational curriculum model for settings with greater curricular maturity and faculty resources.

**How to use this guidance**
Faculties may use this framework to:

1. identify a feasible local starting point,
2. align existing teaching formats with the national core outcomes,
3. plan stepwise curricular expansion over time,
4. avoid overloading the core framework with specialist-level requirements.

**Implementation note**
The bandwidth model should be used as a **feasibility-oriented planning aid**, not as an institutional ranking instrument and not as a substitute for local curriculum mapping. It supports local adaptation while maintaining a shared national educational core.

**Supplementary Material 2. Tumor board teaching package for undergraduate radiation oncology education**

**Purpose**

This teaching package operationalizes the tumor board component of the white paper for undergraduate radiation oncology education. It is intended to support local curricular implementation of the tumor board as an integrative teaching format across different levels of intensity, from a low-threshold seminar-based introduction to more advanced multi-session formats combining simulation, observation, documentation, and reflection.

**Core learning objective**

Students should be able to **explain and critically appraise interdisciplinary decision-making processes** and **understand the function and workflow of a tumor board** in modern cancer care.

**Why this format matters**

The tumor board is particularly suitable as an integrative teaching format because it combines several competencies within a single educational setting:

- clinical reasoning
- evidence-based recommendation building
- interdisciplinary and interprofessional communication
- patient-related trade-off thinking
- assumption of the physician role and responsibility
- prioritization of next steps in cancer care

**Core participant roles and quality criteria**

The format is structured around explicit participant roles. Depending on local resources, these roles may be taken by students, faculty members, or simulated role cards.

Chair
Moderates the discussion, structures the sequence of contributions, ensures time management, and guides the group toward a clear, consensual recommendation.

Presenter
Introduces the case efficiently and in a question-driven manner, including the relevant clinical problem, staging, prior treatment, and the precise tumor-board question.

Radiology
Provides weighted interpretation of imaging findings and explains their relevance for decision-making.

Pathology
Clarifies diagnostic classification and explains its implications for further diagnostics and treatment.

Surgery
Assesses operability and frames procedural trade-offs.

Medical oncology
Names and appraises systemic treatment options, including risk-benefit considerations.

Radiation oncology
Frames indication logic and formulates realistic treatment goals.

Nursing and/or other professions
Highlights supportive care needs, symptom control, treatment limitations, and complexity of care.

The roles are provided with instructional scaffolding, such as role cards, prompts, sentence frames, or brief visual aids.

**General quality criteria for a student tumor board**

A high-quality student tumor board should include:

- a clear clinical question
- concise and relevant case presentation
- discipline-specific contributions that are consequence-oriented rather than merely descriptive
- explicit reasoning about benefits, risks, and alternatives
- visibility of evidence, uncertainty, and prioritization
- a final recommendation that makes next steps, patient preferences, and contingency planning explicit

**Simulation and real-board observation**

The teaching package explicitly values both **simulation** and **real-board observation**.

**What remains similar**

- overall workflow
- interdisciplinary communication
- contribution of multiple professions and specialties

**What differs**

- time pressure
- expert knowledge and expert role
- hierarchical structure of the real board

This comparison is educationally valuable because it helps students distinguish between the didactic clarity of simulation and the authentic complexity of real clinical decision-making.

**Implementation across bandwidths**

**Minimal profile**

**Format**
One session introducing the purpose, workflow, and participant roles of the tumor board, followed by a seminar-based simulation using real or adapted case material.

**Suggested group size**
Flexible. This may be delivered in a large lecture format or in smaller groups of approximately 15–20 students. A flipped-classroom preparation with prior role allocation and shared case material may be used where feasible. At least four roles should be represented.

**Suggested session flow (90–120 minutes)**

- **0–15 min:** introduction to purpose, workflow, and roles
- **15–45/60 min:** teacher-led tumor board simulation
- **45/60–75/90 min:** reflection on the board decision, optionally linked to evidence-based medicine
- **75/90–120 min:** feedback, questions, and brief evaluation

**Recommended profile**

**Format**
At least two sessions, each approximately 90–120 minutes, combining attendance at a real tumor board with a student tumor board simulation.

**Suggested group size and structure**

- 15–20 students
- approximately 5 roles
- 3–5 cases for observation and/or simulation

**Educational focus**
Students should not only observe workflow, but also practice translating complex interdisciplinary information into a structured recommendation.

**Best-practice profile**

**Format**
Four to five sessions, each approximately 90 minutes, with a sequenced progression from preparation to observation, simulation, documentation, and reflection.

**Suggested sequence**

1. **Introduction and self-study preparation**
   Introduction to the tumor board, roles, and preparatory learning materials.
2. **Role allocation and observation tasks for a real tumor board**
   Students receive observation tasks and attend a real board.
3. **Debrief and expert discussion**
   Evaluation of the observed board and discussion of the case material with faculty.
4. **Student tumor board**
   Student-led discussion of the cases, including documentation of the final board decision.
5. **Reflection, feedback, and evaluation**
   Structured debriefing of communication, reasoning, workflow, and interprofessional aspects.

**Suggested teaching workflow for educators**

Educators may use the following sequence as a generic structure:

1. introduce the case and the tumor-board question
2. assign or activate participant roles
3. structure specialist input in sequence
4. encourage explicit discussion of trade-offs and alternatives
5. formulate a final recommendation
6. debrief on communication, hierarchy, evidence use, and decision quality

**Suggested materials**

Where available, faculties may support the format through:

- role cards
- short briefing sheets
- shared case material
- cloud- or Moodle-based preparatory documents
- observation tasks for real-board attendance
- short evaluation forms for reflection and feedback

**Practical implementation note**

Within the white paper, the tumor board is presented as a **particularly suitable integrative teaching format**, not as a mandatory national standard for every site. Faculties may therefore adapt session number, group size, degree of realism, and depth of role differentiation according to local resources and curricular maturity. The key aim is not to reproduce a perfect tumor board, but to make interdisciplinary reasoning, communication, and decision-making visible and learnable for undergraduate students.

**Illustrative example: neuro-oncological tumor board simulation**

**Purpose of the example**
The following worked example illustrates how the tumor board format may be operationalized in undergraduate radiation therapy education at the recommended or best-practice implementation level. It is intended as a concrete use case linking case preparation, interdisciplinary discussion, selected core outcomes, possible assessment formats, and optional AI-supported learning activities.

**Clinical scenario**
An 85-year-old woman with a history of resected pancreatic cancer and prior adjuvant radiochemotherapy presents with newly progressive left-sided weakness. Cerebral imaging suggests a right frontal hemorrhagic brain metastasis with perifocal edema. Her current Karnofsky Performance Status is 70%. The patient has a history of prior pulmonary metastasis, secondary stroke prevention with acetylsalicylic acid, and relevant psychosocial stressors. The interdisciplinary tumor board question is whether local therapy should primarily involve neurosurgical resection, radiotherapy, supportive measures, or a staged multimodal approach.

**Preparation phase (before the session)**
Students receive a short structured case summary including:

- relevant history and oncologic timeline
- neurological status and performance status
- key imaging findings
- pathology and staging information
- a concise tumor-board question
- selected guideline excerpts or key literature

Students are assigned different professional perspectives, for example:

- chair/moderator
- presenting physician
- radiology
- pathology
- neurosurgery
- radiation oncology
- medical oncology/internal medicine
- neurology
- nursing/supportive care

**Tumor board simulation**
Students present the case and discuss:

- the most likely diagnosis and remaining differential considerations
- urgency and prioritization of next diagnostic and therapeutic steps
- arguments for and against neurosurgical resection
- whether radiotherapy may be indicated before or after surgery, or as a non-surgical local treatment option
- symptomatic management, including edema-related measures, antiplatelet considerations, and supportive care
- the role of performance status, prior systemic disease course, and patient-centred treatment goals
- formulation of a final interdisciplinary recommendation, including contingency planning and areas of uncertainty

**Targeted core outcomes**
This example can be used to operationalize several prioritized core outcomes of the framework, especially:

- identification of patients requiring radiation therapy input within multimodal care pathways
- patient-centred benefit-risk communication and basic informed counselling
- recognition and prioritization of oncologic emergencies with relevance to radiotherapy
- formulation of interdisciplinary oncologic treatment recommendations involving radiotherapy
- explanation and critical appraisal of interdisciplinary decision-making processes

**Assessment options**

**Level A (knows how)**
Examples:

- short-answer question on the indication logic for neurosurgical versus radiotherapeutic local treatment
- key-feature question on prioritization of next steps in a patient with suspected brain metastasis and neurological deficit
- brief written case-based question on symptomatic management and referral logic

**Level B (shows how)**
Examples:

- OSCE-type structured case presentation in the role of radiation oncology or chair
- observed five-minute explanation of treatment options and trade-offs in patient-centred language
- structured simulated tumor board participation with rubric-based assessment of reasoning, communication, and recommendation building

**Level C (does, under supervision)**
Examples:

- Mini-CEX or related workplace-based assessment during real or near-authentic tumor board observation
- supervised documentation of a concise tumor board note
- brief observed participation in case discussion during clinical placement with structured feedback

**Optional AI integration under defined conditions**
Students may use generative AI tools in a limited and explicitly supervised way, for example:

- to structure learning questions before the session
- to generate a first draft of a case summary for study purposes
- to compare different ways of formulating the tumor-board question
- to reflect on whether an AI-generated summary omits clinically relevant uncertainties or supportive-care aspects

All AI-supported activities must remain consistent with the framework’s red lines:

- no entry of patient-identifiable data
- no unvalidated medical content without expert review
- transparent disclosure where relevant
- no substitution of human responsibility, expert review, or assessment governance

**Implementation note**
This worked example is intended as an illustrative template, not as a mandatory national case. Faculties may adapt tumor entity, complexity, number of represented professions, session length, and assessment depth according to local curricular context and resources.

**Supplementary Material 3. A/B/C assessment blueprint for undergraduate radiation oncology education**

**Purpose**

This supplementary material translates the staged assessment model of the white paper into a practical planning aid for undergraduate radiation oncology education. It is intended to support faculties in aligning core outcomes with feasible assessment formats while preserving developmental pathways for more advanced observational assessment. The examples below are illustrative implementation formats rather than mandatory national templates.

**Core principle**

The blueprint follows a three-level **A/B/C model** aligned with the Miller pyramid. Its central logic is that every prioritized core outcome should be assessable at a feasible minimum level, while more resource-intensive observational formats may be added where local curricular maturity, patient contact, and faculty resources allow. Within this logic, **Level A** represents the minimum national assessment expectation, whereas **Levels B and C** function as developmental extensions.

**The three levels**

**Level A: applied knowledge (“knows how”)**
Level A uses low-threshold written formats such as short-answer questions, key-feature questions, or brief case-based multiple-choice items. These formats are suitable for securing the minimum standard across heterogeneous sites and should be linked explicitly to outcome-specific blueprints and model answers.

**Level B: observable performance (“shows how”)**
Level B adds structured observational formats, especially OSCE-type stations. In radiation oncology, these formats are particularly suitable for counselling tasks, toxicity communication, or structured case presentation and recommendation.

**Level C: performance under supervision (“does”)**
Level C refers to workplace-based assessment formats such as Mini-CEX or related WBA approaches in simulation, observation, or supervised clinical placement. These formats are intended for settings in which students can be observed briefly during real or near-authentic clinical work and receive structured feedback.

**Quality requirements by level**

**Level A**

- outcome-specific blueprint
- model answer or answer key
- emphasis on clinical reasoning rather than factual recall alone

**Level B**

- clear station prompt
- structured checklist or scoring rubric
- examiner briefing
- explicit marking criteria for observable performance

**Level C**

- structured observation
- mandatory feedback
- entrustment-oriented judgement or equivalent structured rating
- brief documentation of next learning steps

**Minimum expectation and developmental pathway**

Within the framework, every core outcome should be linked to at least one feasible **A-level** format. **B-level** and **C-level** formats are not framed as mandatory national requirements, but as developmental pathways that may be implemented where curricular structures and local resources permit. This distinction is intended to preserve political and curricular feasibility while still encouraging richer observational assessment formats in recommended or best-practice profiles.

**Exemplar B-level OSCE format**

**Educational focus**

The OSCE format is particularly suitable for assessing foundational and intermediate competencies in radiation oncology at **depth levels A–B**, especially in the domain of patient-centred communication and conceptual understanding.

**Addressed core outcome**

Students should be able to place radiotherapy in the overall oncological treatment context, differentiate between curative and palliative treatment goals, and communicate these aspects clearly in a shared decision-making framework. They should further be able to explain basic physical principles of radiotherapy, outline the standard treatment workflow, and describe acute and late toxicities with correct temporal attribution in a patient-centred manner.

**Concept and structure**

A radiation oncology OSCE station simulates a focused segment of a physician–patient counselling encounter. The purpose is to assess whether the student can communicate complex radiation oncology content in a structured, medically accurate, and patient-appropriate way. Each station may be scored using a structured **10-point checklist and a global rating scale**.

**Illustrative station 1: Radiotherapy workflow**

**Task for the student:**
Conduct a counselling conversation and explain the overall radiotherapy process from first consultation to follow-up. The explanation should include informed consent, planning CT, treatment planning, treatment delivery including fractionation, on-treatment monitoring, treatment completion, and follow-up care. The student should use clear and patient-appropriate language.

**Illustrative scoring domains (10 points):**

1. structured overview
2. informed consent mentioned
3. planning CT explained
4. treatment planning explained
5. treatment delivery explained
6. fractionation explained
7. monitoring during radiotherapy addressed
8. treatment completion addressed
9. follow-up mentioned
10. overall explanation understandable and patient-appropriate

**Illustrative station 2: Side effects in breast cancer**

**Task for the student:**
Explain two typical acute side effects and one possible late effect of radiotherapy for breast cancer. The student should distinguish acute from late toxicity and explain practical supportive management options in understandable language.

**Illustrative scoring domains (10 points):**

1. acute toxicity 1 identified
2. acute toxicity 2 identified
3. correct temporal attribution of acute toxicities
4. patient-appropriate explanation
5. supportive management for toxicity 1 proposed
6. supportive management for toxicity 1 made concrete
7. supportive management for toxicity 2 proposed
8. supportive management for toxicity 2 made concrete
9. late effect identified
10. correct temporal attribution of late effect

**Educational interpretation**

These OSCE examples illustrate how core outcomes can move from written minimum assessment to observable performance assessment. They are particularly suitable for recommended or best-practice implementation profiles and may be adapted to local case material.

In addition to OSCE formats, structured oral examinations could offer a resource-efficient alternative, particularly for assessing clinical reasoning and decision-making logic.

**Exemplar B/C-level workplace-based assessment (WBA)**

**Educational focus**

The workplace-based assessment format complements OSCEs by assessing clinical performance in more authentic settings and at greater depth, corresponding to **depth levels B–C**. In radiation oncology, such a format may simulate or accompany a structured ward-round or supervised patient review during the practical year (PJ).

**Concept and structure**

A radiation oncology WBA evaluates whether the student can assess an oncology patient in a structured manner and integrate radiation oncology-specific considerations into diagnostic reasoning, supportive care, and further oncological planning. The expected level of performance is framed at the level of a first-day resident, independent of later specialty choice. Each encounter may be rated using a structured **10-point scale**.

**Illustrative WBA scenario**

**Setting:**
Supervised review of a patient during clinical placement or PJ, for example in a ward-round or supportive-care context.

**Task for the student:**
Perform a focused, structured clinical review of the patient, identify relevant radiation oncology-related aspects, assess acute treatment-related problems, suggest supportive measures, and formulate a coherent next-step plan.

**Illustrative scoring domains (10 points):**

1. structured history taking
2. relevant radiation oncology-specific history
3. focused physical examination
4. interpretation of relevant clinical data, for example laboratory values
5. assessment of acute treatment-related toxicities
6. proposal of supportive measures
7. oncologically meaningful further procedure
8. interprofessional thinking, for example involving nursing or medical oncology
9. professional communication and conduct
10. efficiency and structure of the clinical encounter

**Educational interpretation**

This WBA example illustrates how the framework can extend beyond controlled performance formats toward supervised clinical participation. It is particularly suited to best-practice implementation profiles or to centres with structured PJ teaching and feedback capacity.

**Integration into curriculum planning**

The combination of written A-level formats, OSCE-type B-level formats, and WBA-type B/C-level formats enables a staged and educationally coherent assessment strategy. Written formats secure the minimum standard; OSCEs make communication and reasoning observable in controlled settings; and WBAs support the assessment of supervised clinical performance in authentic or near-authentic contexts. Together, these formats help align outcome orientation, teaching formats, and assessment within a competency-based curriculum.

**Practical implementation note**

This blueprint should be used as a **planning framework** rather than as a rigid national testing prescription. The included OSCE and WBA examples are intended as worked examples to support local curriculum development, blueprinting, and faculty discussion.

**Supplementary Material 4. AI guidance for educators in undergraduate radiation oncology education**

**Purpose**

This guidance supports educators in using generative artificial intelligence (AI) responsibly within undergraduate radiation oncology education. It translates the AI component of the white paper into a practical implementation aid for everyday teaching decisions.

**Core principle**

In this framework, generative AI is understood as both a **teaching and learning aid** and an **explicit learning object**. It may support educational processes under defined conditions, but it does **not** replace human responsibility, disciplinary validation, or assessment governance.

**Permitted under conditions**

AI use may be appropriate in the following educational contexts, provided that transparency, human review, and plausibility checks are ensured:

- **Formative case-vignette drafting**
  AI may support the drafting of teaching cases for seminars or discussion formats, provided that all content is reviewed and adapted by educators.
- **Learning support for structuring and summarizing content**
  AI may be used to help learners organize material, generate summaries, or prepare first drafts for study purposes.
- **AI-assisted drafting of assessment items or rubrics**
  AI may support the drafting of questions, answer keys, or rubrics, but only where blueprinting, disciplinary review, and quality control remain fully human-led.
- **AI literacy as an explicit teaching goal**
  AI itself may be used as an object of instruction, for example when discussing strengths, limitations, hallucinations, bias, transparency, and validation strategies.

**Red lines**

The following rules apply irrespective of local implementation format:

- **No entry of personal patient data** into generative AI tools
- **No use of unvalidated medical content** without source-checking and contextual review
- **Transparent documentation** of tool use, purpose, and scope where relevant
- **Assessment integrity must be defined at the format level**
- **Final responsibility remains human** in all teaching and assessment decisions

**The three-question check for educators**

Before using AI in teaching, educators should ask:

1. **Are the data non-sensitive?**
   No identifiable or sensitive patient information should be entered.
2. **Is the use transparent?**
   Students and faculty should know when AI has been used and for what purpose.
3. **Is disciplinary validation secured?**
   All medically relevant content must be checked for plausibility, accuracy, and contextual appropriateness.

**AI literacy goals for students**

Within the white paper, AI literacy is treated as a small but explicit competency block. Students should be able to:

- explain opportunities and limitations of generative AI in clinical contexts
- identify hallucinations and bias and apply validation strategies
- implement data protection and transparency in practice
- reflect on AI use in ways that consciously address risks such as deskilling

**How educators can use this guidance**

This guidance can be used:

- when preparing AI-supported seminar material
- when designing formative cases or draft assessment items
- when setting local rules for disclosure and review
- when introducing AI literacy into radiation oncology teaching
- when deciding whether a planned AI use is acceptable, questionable, or inappropriate

**Practical implementation note**

The purpose of this guidance is not to encourage unrestricted AI use, but to support **responsible, transparent, and educationally meaningful use under conditions**. In this framework, innovation is tied to governance, and efficiency gains never replace disciplinary responsibility.

**Supplementary Material 5. Preliminary candidate outcomes**

**Purpose**

This supplementary table documents the 15 preliminary candidate outcomes synthesized during the preparatory phase derived from submitted teaching portfolios and used as the starting point for workshop prioritization and consolidation.

| **Supplementary Table 1. Preliminary set of 15 candidate outcomes considered during the preparatory phase** | | |
| --- | --- | --- |
| **No.** | **Candidate outcome** | **Indicative observable performance** |
| 1 | Explain the role of radiation oncology as a major treatment pillar in curative, palliative, and multimodal care | Explains within 60 seconds when radiotherapy is typically used with curative intent and when it is used for symptom-oriented or palliative purposes |
| 2 | Identify patients who require radiation oncology review and formulate an appropriate referral question | Formulates a precise consultation question and gives two reasons why radiation oncology input is indicated |
| 3 | Describe the radiation oncology workflow from first contact to follow-up | Produces a five-step process map covering first contact, simulation/planning, fractionation, treatment, and follow-up, including one potential risk or pitfall for each step |
| 4 | Apply basic principles of radiation protection and safety measures | Names three core principles of radiation protection and derives one relevant clinical consequence |
| 5 | Recognize and initiate management of acute toxicities in common radiotherapy scenarios | Identifies red flags and initial management steps in two typical toxicity scenarios |
| 6 | Outline typical late effects and sketch basic prevention and follow-up logic | Names two typical late effects and two relevant follow-up aspects |
| 7 | Recognize and prioritize oncologic emergencies with potential relevance to radiotherapy | Prioritizes an acute management pathway in three steps |
| 8 | Justify an interdisciplinary treatment recommendation | Formulates a treatment recommendation, a contingency plan, and one clearly stated uncertainty |
| 9 | Explain the logic of evidence and guideline-based decision-making | Names one anchor of evidence and one relevant limitation |
| 10 | Conduct a brief principle-based informed counselling conversation | Conducts a structured five-minute counselling conversation at a basic principle level |
| 11 | Demonstrate patient-centred benefit-risk communication | Identifies relevant trade-offs and actively elicits patient preferences |
| 12 | Explain palliative treatment options including radiotherapy | Explains treatment options ranging from prophylactic to palliative settings, including potential risks |
| 13 | Document a tumor board note or treatment plan in a concise and action-oriented way | Writes a clear and action-oriented board note in six lines |
| 14 | Explain the roles of other professions and teamwork along the oncologic care pathway | Identifies roles, responsibilities, and key interfaces between professions |
| 15 | Demonstrate basic AI competence relevant to study and clinical transfer | Makes transparency, validation, and data protection visible in a practical way |
| Note: This table reflects the preliminary candidate outcome set prior to workshop prioritization. | | |
